# Assessment of a new protocol strategy to control the ectoparasitic infestation in Nile tilapia (*Oreochromis niloticus*) using efficient natural products

**DOI:** 10.1186/s12917-024-04387-z

**Published:** 2025-01-11

**Authors:** Magdy I. Hanna, Aya T. EL Sayed, Ola Hasan Abd El Megeed, Marwa A. Ibrahim, Reda M. S. Korany, Marwa M. Attia

**Affiliations:** 1 Department of Aquatic Animal Medicine and Management, Faculty of Veterinary Medicine, Cairo University, Giza, 12211 Egypt; 2 Department of Biochemistry and Molecular Biology, Faculty of Veterinary Medicine, Cairo University, Giza, 12211 Egypt; 3 Department of Pathology, Faculty of Veterinary Medicine, Cairo University, Giza, 12211 Egypt; 4 Department of Pathology, Faculty of Veterinary Medicine, Egyptian Chinese University, Cairo, Egypt; 5 Department of Parasitology, Faculty of Veterinary Medicine, Cairo University, Giza, 12211 Egypt

**Keywords:** External parasites, Herbal treatment, Ectoparasites, *Oreochromus niloticus*, Monogenean

## Abstract

This study aimed to evaluate alternative in vivo treatment trials using natural products for ectoparasitic infestation on Nile tilapia; these two products were not previously used in the treatment of parasitic fish diseases. So, a total of 400 *Oreochromis niloticus (O. niloticus)* fish measured 10–15 cm in length; 350 from a fish farm in (Kafr Elsheikh and 50 from Nile River (Al Bahr Al Aazam), Egypt. The examined fishes were 10–15 ± 0.5 cm long and weighed from 45 g ± 5. The collected fish were examined for different clinical abnormalities. Each part of the fish underwent a careful microscopic examination of mucous surrounding the skin; gills and fins. Two feed supplements were used experimentally to decrease mortality and treat fish against ectoparasites (Herb-All PARA-X^®^ and Herb-All CALM^®^). Total mRNA was extracted from the gills of different examined groups. Glucose; nitric oxide; cortisol as well as lysozyme activity were assessed in all groups. The gills of the examined fish were collected for histopathological examination. Only, *Dactyolgyrus* sp. was recovered. The intensity of the parasite was counted per microscopic field. The treated groups showed low levels of the parameters compared to the control positive group. Up-regulation of both Tumor necrosis factor-α (TNF- α) and heat shock protein 70 (hsp-70) were detected in fins, gills, and skin in the infested tilapia. The treatment and prophylaxis significantly downregulated both genes in the studied organ in a dose-dependent manner. Recorded lesions which were scored according to their severity. In conclusion; following the use of those products, fish health has been greatly improved and that is indicated by findings of immune reactions as well as histopathology.

**Supplementary Information:**

The online version contains supplementary material available at 10.1186/s12917-024-04387-z.

## Introduction

Parasites grown in culture conditions proliferate rapidly and cause severe outbreaks. This is especially true for monogeneans and protozoans that have a direct life cycle which can be easily finished in a closed environment and don’t have intermediate hosts [1]; [2]. The most prevalent ciliates seen on the skin and gills of fish raised in ponds are species of the genus *Trichodina* [3]; [4]. Fish with an infestation struggle to eat, get weaker, and are therefore more vulnerable to opportunistic bacterial diseases in the water. Numerous investigations have documented monogenea species infestations in Nile tilapia [5–7]. More than 100 families make up the monogeneans, and the majority of the species are host and location-specific, meaning they only need one host to complete their life cycle. Numerous species of monogeneans have been linked to the demise of tilapia, both in the wild and in culture [8]. Monogenean parasites can infest the skin and gills of both freshwater and marine fish, and they have a single-host life cycle [9]; [10].

Hatcheries often use the therapeutic dosages of chemical disinfectants—currently just formalin, sodium chloride, hydrogen peroxide, and acetic acid—to control ectoparasites (11–12). There are two types of treatments for monogenean infestation: preventative and curative. While treatment can be given orally or by bathing, prophylactic treatments are usually given orally. Prophylactic; curative and oral treatments are favored over bath treatments because they don’t require additional infrastructure or animal handling [13]. Additionally, administering in-feed treatment is quicker and involves going straight to the host rather than the host’s surroundings. Because they prevent the strains brought on by parasite infestations, preventive treatments are better than curative ones [14]. The problems with food safety brought on by chemotherapy agent residues must also be taken into account [15]. Research on alternate prophylactics to fight monogenean infestation in aquaculture has been spurred by these eco-friendly components and other unfavorable side effects of chemotherapy treatments [16]. Therefore; this study aimed to evaluate alternative eco-friendly herbal extract; Herb-All PARA-X^®^ (contains *Curcuma longa; Allium sativum* and the carrier was nut hulls) while Herb-All CALM^®^ (contains *Ocimum sanctum; Terminalis bellerica* and the carrier was nut hulls). These two products were produced by Life Circle Nutrition AG (Wangen/Switzerland). Which is not previously used in the treatment of ectoparasites in fish on Nile tilapia with an assessment of these trials using oxidative stress markers; gene expression analysis and histopathological studies of different examined groups.

## Materials & methods

### Collection of samples

A total of 400 *Oreochromis niloticus (O. niloticus)* fish measured 10–15 ± 0.5 cm in length and weighing from 40 to 50 g ± 5); (350 from a fish farm in Kafr Elsheikh and 50 from Nile River; Al Bahr Al Aazam), Egypt. These fish were surveyed for ectoparasites. The fish were brought alive and transported in plastic containers supplied with oxygen to the Laboratory of Aquatic Animals Diseases and management, faculty of Veterinary Medicine, Cairo University for clinical, parasitological; histopathological, and immunological analysis. All fish were kept in several aerated covered glass aquaria of 60 L capacities with 15–20 fish per aquaria.

### Clinical examination of fish

Collected fish were examined for any clinical abnormalities according to the method described by **Amlacher** [17]. To make it easier to scrape off the skin, fins, and gills, the fishes were euthanized with an overdose of commercial clove oil 0.5 ml/l (Ectyo-colve^®^, France).

### Parasitological examination of fish

Each part of the fish (one hundred fishes were examined for parasitological analysis; Attia et al. [7]; [18] was examined carefully under the light microscope (mucous surrounding the skin; gills and fins); from each examined part a fixed smear with methanol was prepared; then these smears were stained with prepared Giemsa stain; Ibrahim et al. [19], as well as all the collected parasites, were identified and photographed according to Yamaguti [20].

### Feed supplements

Two feed supplements were used for experimental design to decrease the mortality and treatment of the fish against external parasites. Herb-All PARA-X^®^ (contains *Curcuma longa; Allium sativum* and the carrier was nut hulls) while Herb-All CALM^®^ (contains *Ocimum sanctum; Terminalis bellerica* and the carrier was nut hulls). These two products were produced by Life Circle Nutrition AG (Wangen/Switzerland). The diet (30% protein) was obtained from a private company (Aqua International; Company; Egypt) for food industries. The diet composed of 30% crude protein; 4100 Kcal/kg total energy; 5.8% crude fat and not less than 7.5% crude fiber 44% soybean, yellow corn and sunflower 28%. Fish pelletized food was covered with 1.0% gelatain and mixed groups with Herb-All PARA-X^®^ and Herb-All CALM ^®^ in a treated aquarium. One hundred and fifty of the examined *O. niloticus* fish were classified into 5 groups (30 fishes for each group, triplicate for each group).

### Experimental trials using Herb-All PARA-X^®^

One hundred and fifty of the examined *O. niloticus* fish were classified into 5 groups (30 fish, triplicate for each group) as follows: G1: the first group was a control negative group: apparent healthy fish receiving an artificial diet without additives. G2: The second group acted as a control positive group: fish infested with ectoparasites receiving an artificial diet without any additives. G3: The third group: fish infested with ectoparasites (either naturally or experimentally infested) receiving an artificial diet mixed with Herb-All PARA-X (1 kg/ton). G4: the fourth group: fish infested with ectoparasites (either naturally or experimentally infested) receiving an artificial diet mixed with Herb-All PARA-X (2 kg/ton). G5: the fifth group: fish infested with ectoparasites (either naturally or experimentally infested) receiving an artificial diet mixed with Herb-All PARA-X (4 kg/ton); **(supplementary file)**. The experiment was run for 4 weeks where fish samples (5 euthanized fish) were collected weekly from each group for weighing of fish samples and calculation of the quantity of artificial diet. Parasitological examinations were applied on skin and gill scrapings (qualitative and quantitative examination). Histopathological studies of fish gills in different groups were examined and analyzed. Serum samples were collected for examination of stress parameters (cortisol and glucose) as well as the immune status of fish (serum lysosomes and nitric oxide assay). Estimation of stress genes for Hsp-70, TNF-α, and gapdh at the first and fourth week.

### Treated trials using Herb-All CALM^®^

The experimental design for Herb-All CALM: *O. niloticus* fish were classified into 3 groups (30 fish in each group) as follows: G6: fish was receiving an artificial diet mixed with Herb-All CALM (1 kg/ton). While G7 was receiving an artificial diet mixed with Herb-All CALM (2 kg/ton). As well as G8 received an artificial diet mixed with Herb-All CALM (4 kg/ton); (supplementary file).

Fish samples were collected in the second and fourth week for: 1- serum sampling to estimate the stress parameters (cortisol and glucose) and for the immune status of fish (serum lysosomes and nitric oxide assay). 3- estimation of stress genes for *Hsp-70*,* and Tnf-a*, at the first and fourth week. 4-estimation of feed conversion ratio was according to [19]; [21].

$${\rm{Feed}}\,{\rm{conversion}}\,{\rm{ratio}}\,{\rm{(FCR)}} = {{total\,feed\,{\rm{int}}ake\,(g)} \over {total\,feed\,gain\,(g)}}$$

The experiment ran for 4 weeks.

5-Estimation of mortality rate according to **Ram and Ram** [22].

$${\rm{Mortality}}\,{\rm{rate = }}{{No.of\,dead\,fish} \over {No.of\,total\,fish}} \times 100$$

#### Transcript levels of the examined genes in the treatment groups

##### Total RNA extraction and cDNA synthesis

Total RNA was extracted from gills using the QIAmp RNA mini kit from Qiagen, following the manufacturer’s protocol. The first strand cDNA was then synthesized using M-MuLV reverse transcriptase); [23].

#### Quantitative real-time PCR (qPCR) for examination of immune fish health status

The PCR reactions were prepared using the iQ SYBR GREEN SUPERMIX from BIO-RAD; this reaction was run in Biochemistry department; Faculty of Veterinary Medicine; Cairo University. The amplification was carried out in the BIO-RAD iCycler thermal cycler and detected using the MyiQ real-time PCR detection system, as described by [24]; [25]. The primers (Table 1) used to amplify the target genes were designed based on the published sequence in the Genbank of *O. niloticus*. The PCR program consisted of an initial pre-incubation step at 95 °C for 10 min, followed by 35 cycles of denaturation at 95 °C for 20 s, annealing at 60 °C for 20 s, and extension at 72 °C for 30 s, as reported by [26]; [27]. Each sample was tested in duplicate, and a no-template negative control was included, following the protocol described by **Attia**et al. [28]. To normalize the expression data, the gene gapdh was used as an internal control, as suggested by **Ko**et al. [29]. Finally, the gene expression data were calculated using the ^2^−ΔΔ^CT method.

**Table 1:** The primer sequences of the target genes

Gene	Formard primer	Reverse primer	Accession number	Amplicon (bp)
***Hsp-70***	AAAGGTGTAGCGATCGGCAT	CCACATAACTGGGGGTGGTC	*XM_003442456.5*	121
***Tnf-a***	GCCTCACAATTCTCAGCCAC	AAACACGCCAAAGAAGGTCC	AY428948.1	248
***gapdh***	GCTGTACATGCACTCCAAGG	ACTCAAACACACTGCTGCTG	*NM_001279552.1*	182

#### Assessment of glucose levels in the treated groups

Glucose was estimated by using a glucose test kit, manufactured by JEEV, India. Diagnostics PVT.LTD and using O.D at 505 nm according to [30]; [31].

#### Evaluation of nitric oxide levels

Nitric oxide was estimated by using Nitric oxide (NO) Colomeritic assay kit, Manufactured by Elabscience, USA. Using O.D at 550 nm according to **Wang**et al. [32].

#### Assessment of cortisol levels in the treated groups

Cortisol was estimated by using an Enzyme immunoassay test kit, manufactured by Perkin Elmer Health Sciences, Inc, USA; [33].

#### Serum lysozyme activity of the treated groups

The activity of serum lysozyme was measured using the protocol of **Eissa et al.** [34]; and **Kumar et al.** [35]. The assay involved mixing of *Micrococcus lysodeikticus* (ATCC No. 4698; Sigma- Aldrich) solution (0.75 mL; pH 6.2, 0.2 mg/mL PBS) with 0.25 mL of serum. The reaction was carried out at room temperature and the absorbance was measured at 450 nm after 20 min using a spectrophotometer (BM Co. Germany). The serum lysozyme concentrations were calculated according to **Ragab et al.** [36].

### Histopathological examination

Gills specimens were collected from different groups, fixed in neutral buffered formalin 10%, washed, dehydrated, cleared, and embedded in paraffin. The paraffin-embedded blocks were sectioned at 5-micron thickness and stained with Hematoxylin and Eosin [37] for histopathological examination by a light microscope (Olympus BX50, Japan).

### Histopathological lesion scoring

Histopathological alterations in gills were recorded and scored as, no changes (0), mild (1; <30%), moderate (2; <30 – 50%) and severe (3; >50%) changes [38].

### Statistical analysis

Minimum, maximum, means, and standard error of means (SEM) have been used to describe the data. Statistical analysis was done using One-Way ANOVA and Independent Sample T-test.

## Results

**Fig. 1:**
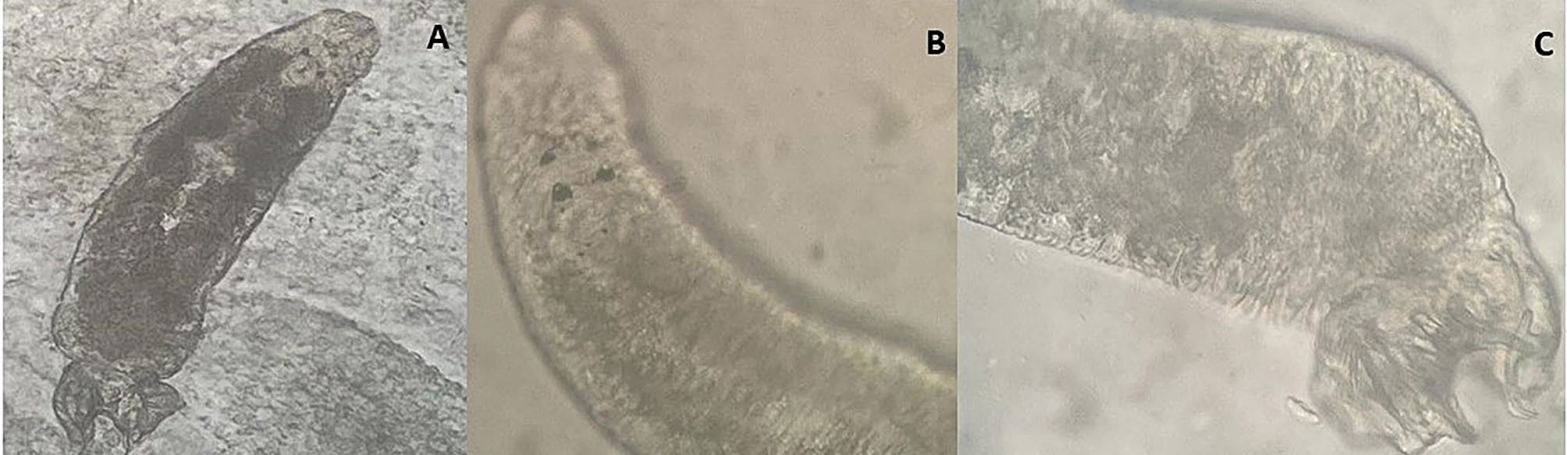
*Dactyolgyrus* sp. infested fish under experimental design. **A**: whole monogenean body; **B**: Anterior end showing the four eyes spots; **C**: Posterior end with opisthapitor

Collected parasites from skin and gills under investigation was *Dactyolgyrus* sp. (Fig. 1) which were decrease during the treatment trials; the intensity of parasites was counted per microscopic field as in first week was 15 ± 2; which decreased gradually by 12 ± 1; 6 ± 1; 2 ± 1 in second; third and fourth week respectively; the G2 (positive control without treatment) showed 16 ± 3.

### Assessment of glucose levels in treatment groups (Table 2)

**Table 2:** Assessment of glucose levels in treatment groups *treatment trials with Herb-All PARA-X* (mg /dL)

Groups	G1	G2	G3	G4	G5	*P*-value
Weeks						
w1	20–23 (21 ± 0.9)^b^	68–72 (70 ± 1)^a, A^	33–60 (46 ± 8)^b^	46–60 (52 ± 4)^b, A^	49–52 (56 ± 6)^b, A^	< 0.001
w2	22–25 (23 ± 0.9)^b^	63–68 (65 ± 1)^a, B^	18–28 (22 ± 3)^b^	19–27 (22 ± 2)^b, B^	18–25 (22 ± 2)^b, B^	< 0.001
w3	20–26 (24 ± 1.9)^b^	52–59 (55 ± 2)^a, B^	15–17 (18 ± 2)^b^	15–25 (19 ± 3)^b, B^	15–24 (19 ± 3)^b, B^	< 0.001
w4	18–26 (23 ± 2)^b^	40–47 (44 ± 2)^a, B^	18–22 (19 ± 1)^b^	14–28 (20 ± 4)^b, B^	17–25 (20 ± 3)^b, B^	< 0.001
*P-*value		< 0.001		< 0.001	< 0.001	

^a, b^ Different superscripts in the same row indicate significant differences at *P* < 0.05

^A, B^ Different superscripts in the same column indicate significant differences at *P* < 0.05

G2 (positive control) showed a high concentration of glucose level in comparison to G1 (negative control). While G3-G8 showed a lower concentration of glucose level than G2 and a higher than G1; Table 3.

**Table 3:** Assessment of glucose levels in prophylaxis groups with Herb-All CALM (mg /dL)

Groups	G1	G2	G6	G7	G8	*P*-value
Weeks						
W2	22–25 (23 ± 0.9)^b^	63–68 (65 ± 1)^a, A^	19–30 (25 ± 3)^b, B^	18–29 (22 ± 3)^b^	17–26 (23 ± 3)^b^	< 0.001
W4	18–26 (23 ± 2)^b^	40–47 (44 ± 2)^b, B^	37–41 (39 ± 1)^a, A^	29–32 (31 ± 1)^b^	23–28 (26 ± 2)^b^	< 0.001
*P-*value		0.001	0.013			

^a, b^ Different superscripts in the same row indicate significant differences at *P* < 0.05

^A, B^ Different superscripts in the same column indicate significant differences at *P* < 0.05

### Evaluation of nitric oxide levels (Tables 4 and 5)

**Table 4:** Evaluation of nitric oxide levels in treatment group (µmol/L)

Groups	G1	G2	G3	G4	G5	*P*-value
Weeks						
W1	6.92-8 (7 ± 0.3)^b^	58.57–64.67 (61 ± 2)^a^	59–63 (60 ± 1)^b, A^	57–61 (59 ± 1)^b, A^	55–59 (57 ± 1)^b, A^	< 0.001
W2	5.71-9 (7 ± 1)^b^	65.78–68.2 (67 ± 0.8)^a^	20–22 (21 ± 0.6)^b, B^	15.71–18.43 (17 ± 0.9)^b, B^	11.8-14.57 (13 ± 0.8)^b, B^	< 0.001
W3	5.23–7.23 (6 ± 0.6)^b^	64-69.6 (67 ± 2)^a^	16–18 (17 ± 0.6)^b, B^	12–15 (14 ± 0.9)^b, B^	9–11 (10 ± 0.6)^b, B^	< 0.001
W4	5.83–7.3 (6 ± 0.4)^b^	63.4–67.6 (66 ± 1)^a^	10–15 (12 ± 0.1)^b, B^	9–11 (10 ± 0.6)^b, B^	8–10 (9 ± 0.6)^b, B^	< 0.001
*P-*value			< 0.001	< 0.001	< 0.001	

^a, b^ Different superscripts in the same row indicate significant differences at *P* < 0.05

^A, B^ Different superscripts in the same column indicate significant differences at *P* < 0.05

**Table 5:** Evaluation of nitric oxide levels in prophylaxis group (µmol/L)

Groups	G1	G2	G6	G7	G8	*P*-value
Weeks						
W2	5.71.9 (7 ± 1)^b^	65.78–68.2 (67 ± 0.8)^a^	16.71–18.57 (18 ± 0.5)^b^	13–16 (14 ± 0.9)^b, A^	10–13 (11 ± 0.9)^b, A^	< 0.001
W4	5.83–7.3 (6 ± 0.5)^b^	63.4–67.6 (66 ± 1)^a^	12–16 (14 ± 0.9)^b^	8–12 (10 ± 1)^b, B^	5–9 (7 ± 1)^b, B^	< 0.001
*P-*value				0.041	0.041	

^a, b^ Different superscripts in the same row indicate significant differences at *P* < 0.05

^A, B^ Different superscripts in the same column indicate significant differences at *P* < 0.05

G2 (positive control) showed a high concentration of nitric oxide in comparison to G1 (negative control). While G3-G5 showed a lower concentration of nitric oxide than G2. G6 to G8 showed a lower concentration of nitric oxide than G2 and a higher than G1; Table 5.

### Cortisol levels of the treatment groups

G2 (positive control) showed a high concentration of cortisol level in comparison to G1 (negative control). While G3-G5 showed a lower concentration of cortisol level than G2; Table 6. The G6 to G8 showed a lower concentration of cortisol level than G2 and a higher than G1; Table 7.

**Table 6:** Cortisol levels of the treatment groups (ng/ml)

Groups	G1	G2	G3	G4	G5	*P*-value
Weeks						
W1	20–28 (24 ± 2)^b^	390–430 (413 ± 12)^b, A^	380–430 (410 ± 15)^b, A^	390–418 (377 ± 34)^b, A^	312–500 (421 ± 56)^a, A^	< 0.001
W2	22–25 (23 ± 2)^b^	320–460 (398 ± 41)^a, B^	17–20 (18.5 ± 0.9)^b, B^	15–30 (24 ± 5)^b, B^	15–18 (16 ± 0.9)^b, B^	< 0.001
W3	20–26 (24 ± 1)^b^	280–410 (360 ± 40)^a, B^	16–27 (21 ± 3)^b, B^	15–23 (20 ± 3)^b, B^	14–17 (15 ± 1)^b, B^	< 0.001
W4	20–25 (23 ± 1)^b^	180–290 (240 ± 32)^a, B^	14–28 (23 ± 4)^b, B^	13–27 (19 ± 4)^b, B^	12–15 (14 ± 0.9)^b, B^	< 0.001
*P-*value		0.024	< 0.001	< 0.001	< 0.001	

^a, b^ Different superscripts in the same row indicate significant differences at *P* < 0.05

^A, B^ Different superscripts in the same column indicate significant differences at *P* < 0.05

**Table 7:** Cortisol levels of the treatment groups (ng/ml)

Groups	G1	G2	G6	G7	G8	*P*-value
Weeks						
W2	22–25 (23 ± 2)^b^	320–460 (398 ± 41)^a^	23.2–26.7 (25 ± 1)^b, B^	23.7–28.7 (26 ± 2)^b, B^	21.9–30.7 (25 ± 3)^b, B^	< 0.001
W4	20–25 (23 ± 1)^b^	180–290 (240 ± 32)^b^	370–410 (393 ± 12)^a, A^	262–320 (287 ± 17)^b, A^	183–220 (201 ± 9)^b, A^	< 0.001
*P-*value			< 0.001	< 0.001	< 0.001	

^a, b^ Different superscripts in the same row indicate significant differences at *P* < 0.05

^A, B^ Different superscripts in the same column indicate significant differences at *P* < 0.05

### Serum lysozyme activity of treatment groups; Table 8

**Table 8:** Serum lysozyme activity of treatment groups (µg/mL)

Groups	G1	G2	G3	G4	G5	*P*-value
Weeks						
W1	92–115 (104 ± 7)^b^	230-259.9 (244 ± 9)^a^	189.75-203.55 (197 ± 4)^b, A^	184-190.9 (187 ± 2)^b, A^	179.4-193.2 (186 ± 4)^b, A^	< 0.001
W2	88–110 (99 ± 6)^b^	220-248.2 (234 ± 8)^a^	181.15–194.7 (188 ± 40^b, B^	176-182.6 (179 ± 2)^b, B^	171.6-181.8 (178 ± 4)^b, B^	< 0.001
W3	84–105 (95 ± 6)^b^	210-237.3 (223 ± 8)^a^	173.25-185.85 (180 ± 4)^b, B^	168-174.3 (171 ± 2)^b, B^	163.8-176.4 (170 ± 4)^b, B^	< 0.001
W4	80–100 (90 ± 6)^b^	200–226 (213 ± 8)^a^	165–177 (171 ± 3)^b, B^	160–166 (163 ± 2)^b, B^	156–168 (162 ± 3)^b, B^	< 0.001
*P-*value			0.007	< 0.001	0.009	

^a, b^ Different superscripts in the same row indicate significant differences at *P* < 0.05

^A, B^ Different superscripts in the same column indicate significant differences at *P* < 0.05

G2 (positive control) showed a high serum lysozyme activity in comparison to G1 (negative control). The G3-G5 showed a lower serum lysozyme activity than G2. As well as G6-G8 showed a lower serum lysozyme activity than G2 and a higher than G1. (Table 9.

**Table 9:** Serum lysozyme activity of prophylaxis groups (µg/mL)

Groups	G1	G2	G6	G7	G8	*P*-value
Weeks						
W2	88–110 (99 ± 6)^b^	220-248.2 (234 ± 8)^a^	93.5-115.5 (105 ± 6)^b^	88-114.4 (101 ± 8)^b^	91.3-113.3 (102 ± 6)^b^	< 0.001
W4	80–100 (90 ± 6)^b^	200–226 (213 ± 8)^a^	85–105 (95 ± 6)^b^	80–104 (92 ± 7)^b^	83–103 (93 ± 6)^b^	< 0.001
*P-*value						

^a, b^ Different superscripts in the same row indicate significant differences at *P* < 0.05

^A, B^ Different superscripts in the same column indicate significant differences at *P* < 0.05

### Transcript levels of the examined genes against treated groups

**Fig. 2:**
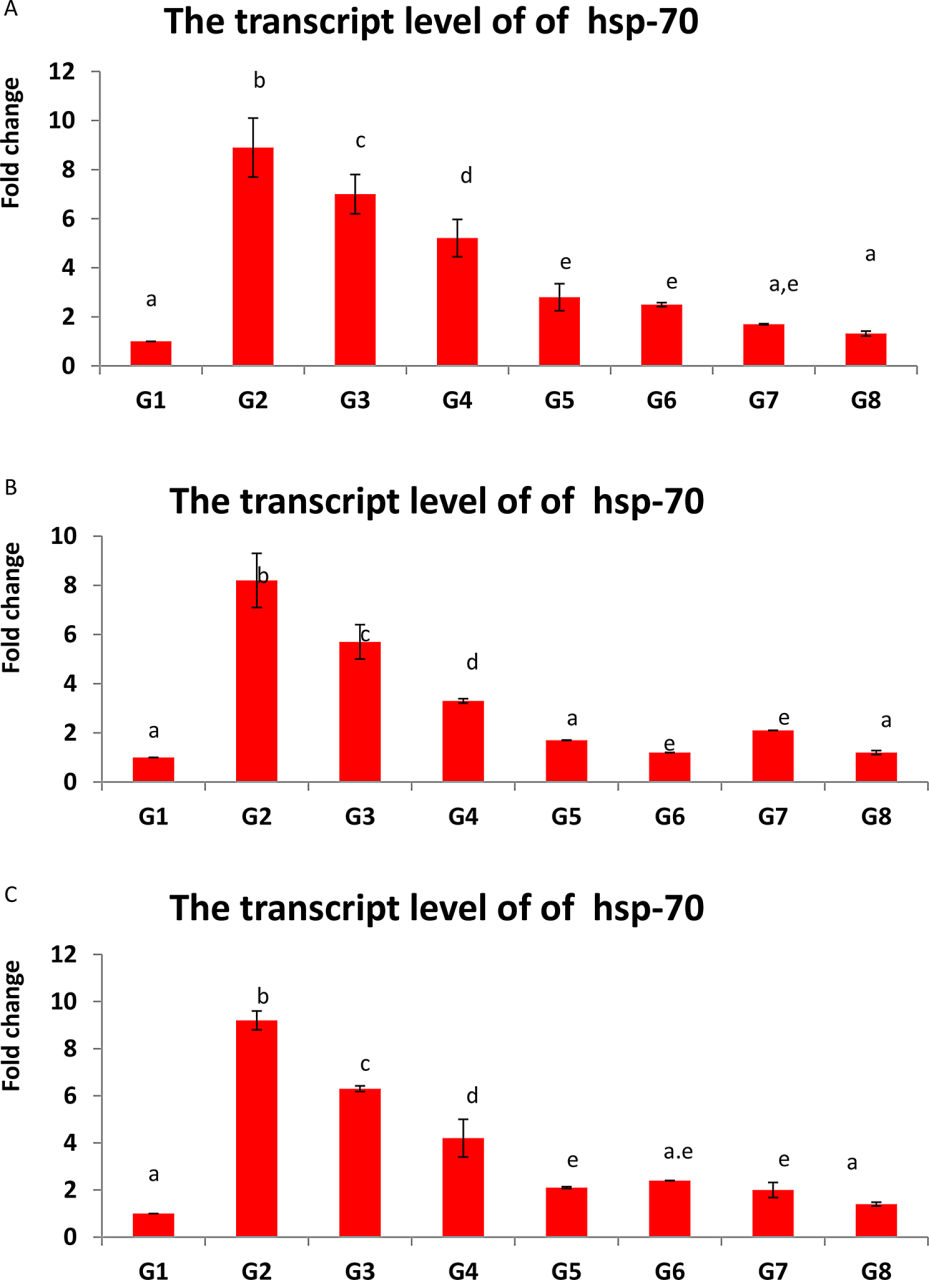
The bar chart of the transcript level of hsp-70 in the (**A**): gills; (**B**) fins; (**C**) skin. Values are presented as mean ± SEM. (*n* = 5 fish/group). Different superscript indicates statistically significant difference at *p* < 0.05

**Fig. 3:**
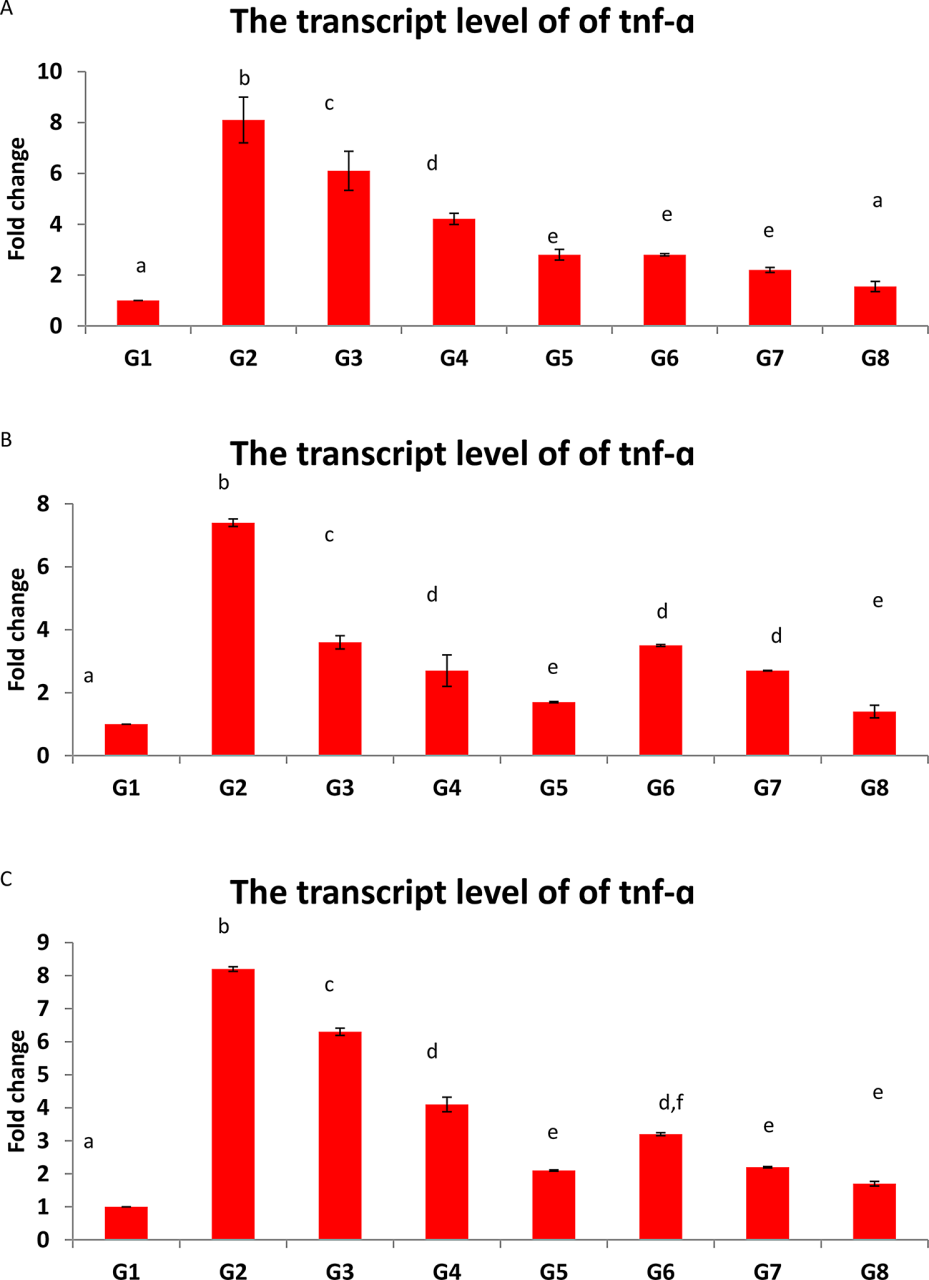
(**B**): The bar chart of the transcript level of TNF-ɑ in the in the (**A**): gills; (**B**) fins; (**C**) skin. Values are presented as mean ± SEM. (*n* = 5 fish/group). Different superscript indicates statistically significant difference at *p* < 0.05

Up-regulation of both TNF-α and hsp-70 were detected in fins, gills and skin in the infested tilapia. The G3, G4,G5,G6,G7 and G8 showed significantly down regulation of both genes in all the studied organs in a dose- dependent manner at *p* < 0.05; (Figs. 2 and 3).

### Histopathological findings

**Fig. 4:**
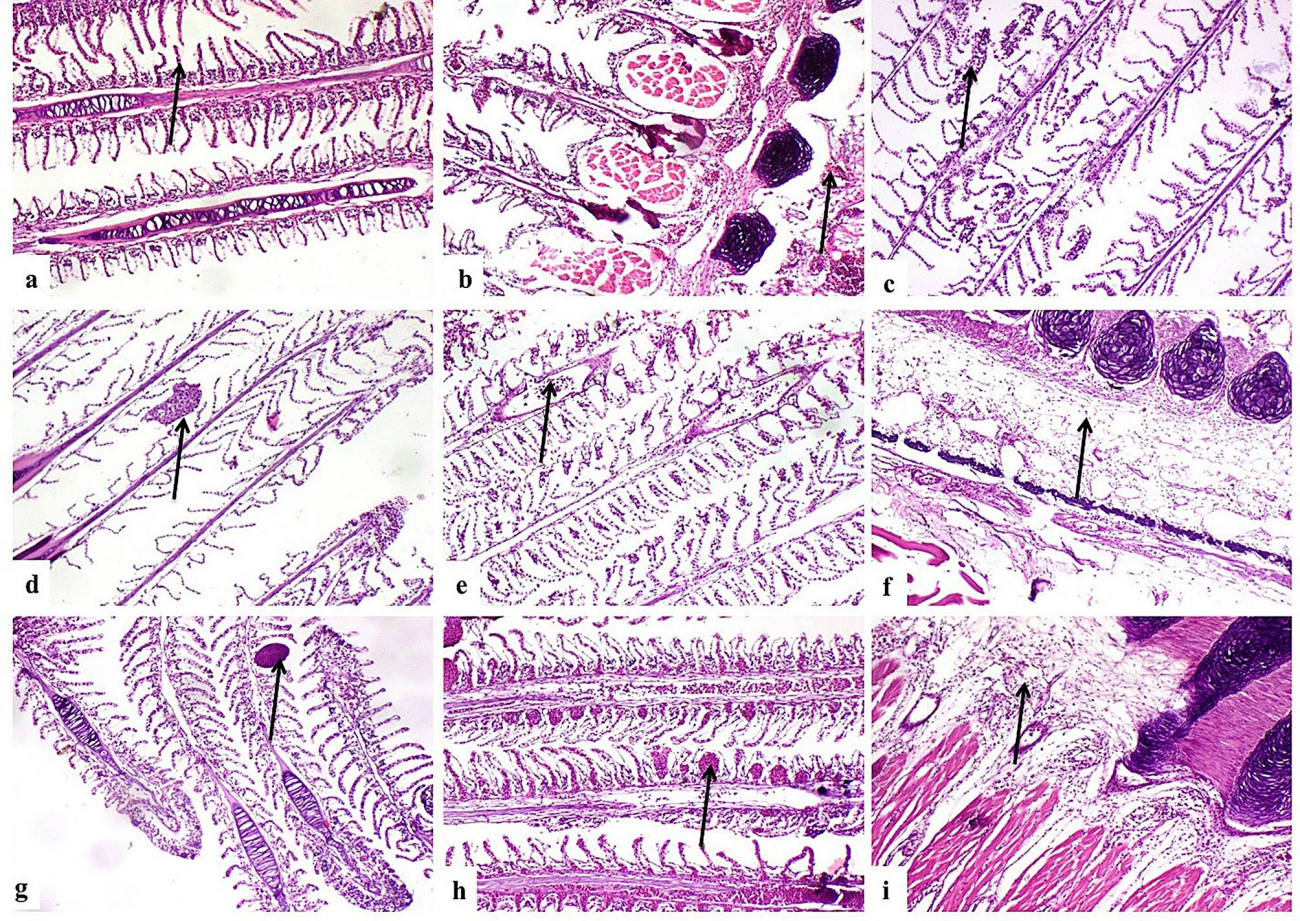
(**a**) G1, normal histological structure of gill lamellae (arrow). (**b**) G1, normal histological structure of gill arch (arrow). (**c**) & (**d**) G2, sections of parasites between gill filaments (arrow). (**e**) G2, congestion of primary gill lamellae blood vessels (arrow). (**f**) G2, edema and inflammatory cells infiltration in gill arch connective tissue (arrow). (**g**) G3, degenerated parasites between gill filaments (arrow). (**h**) G3, lamellar telangiectasis (arrow). (**i**) G3, edema and inflammatory cells infiltration in gill arch connective tissue (arrow).(H&EX200)

**Fig. 5:**
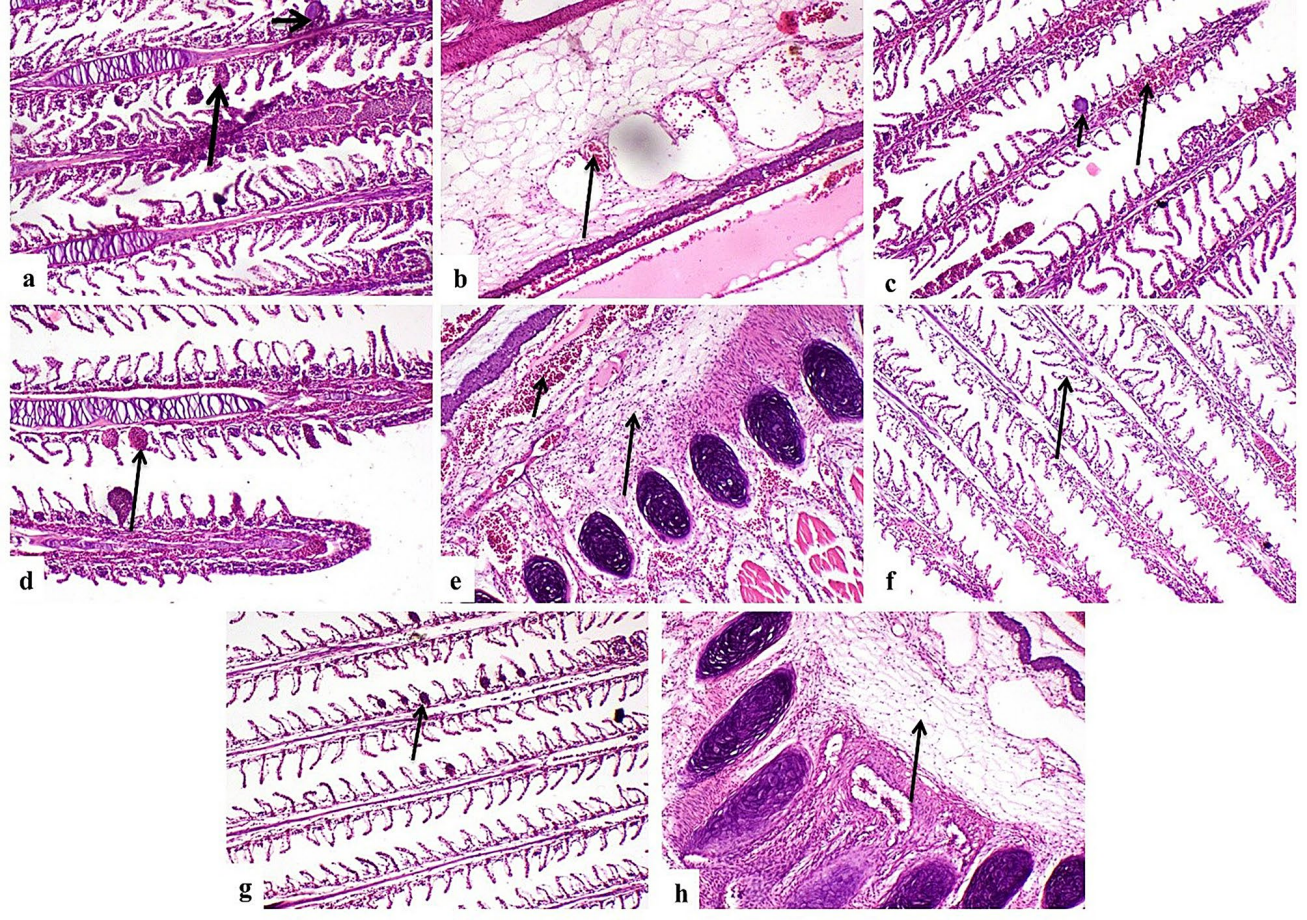
(**a**) G4, degenerated parasitic sections (short arrow) and moderate lamellar telangiectasis (long arrow). (**b**) G4, moderate edema and congestion of gill arch (arrow). (**c**) G5, degenerated parasite (short arrow), congestion of primary gill lamellae blood vessels (long arrow). (**d**) G5, moderate lamellar telangiectasis (arrow) (**e**) G5, edema, congestion (short arrow) and inflammatory cells infiltration in gill arch (long arrow). (**f**) G6, absence of parasites between gill filaments (arrow) (**g**) G6, mild lamellar telangiectasis (arrow). (**h**) G6, mild edema in gill arch connective tissue (arrow). (H&EX200)

**Fig. 6:**
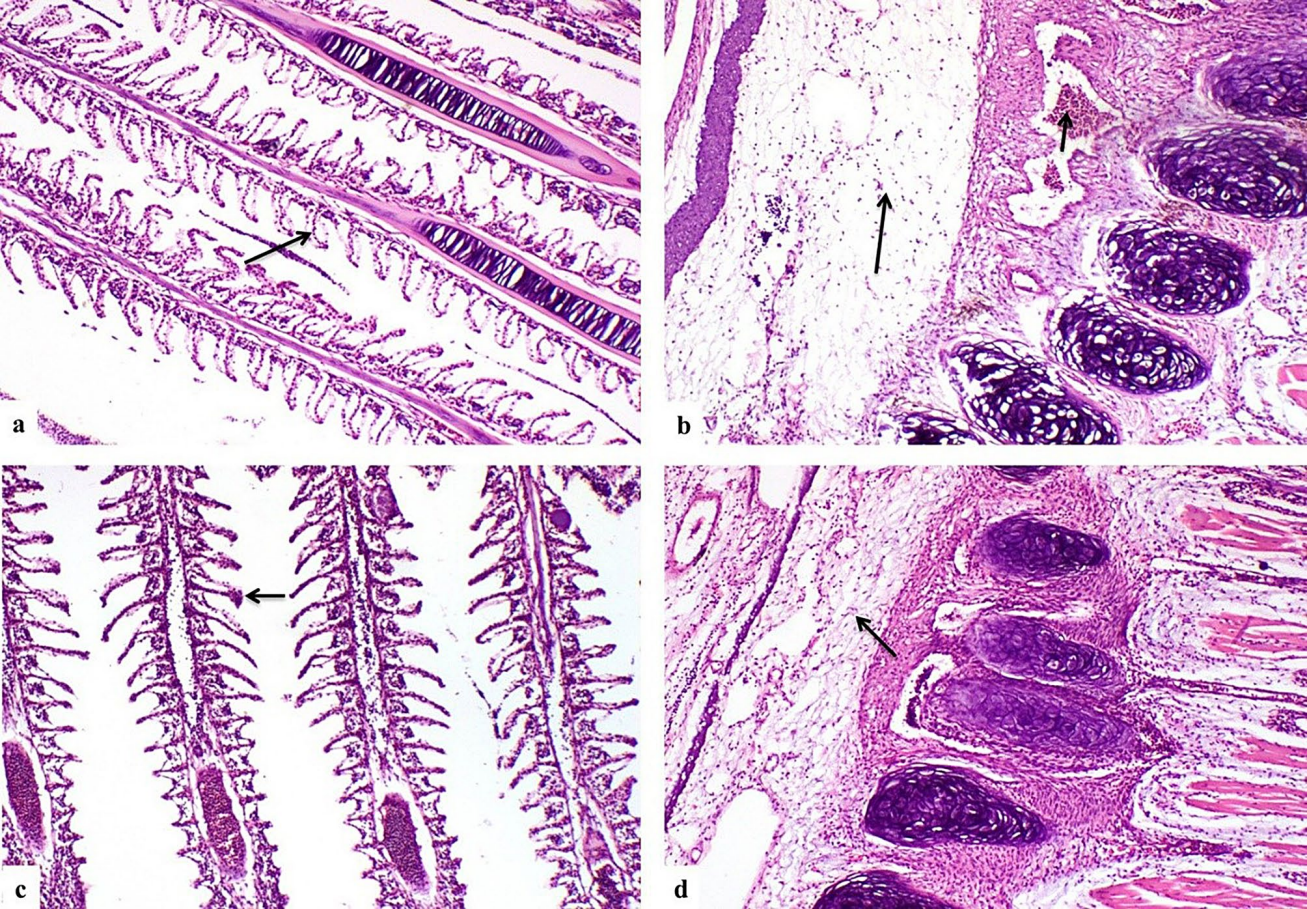
(**a**) G7 nearly normal gill lamellae (arrow) (**b**) G7,edema in gill arch connective tissue (arrow). (**c**) G8, mild lamellar telangiectasis (arrow) (**d**) G8, mild gill arch connective tissue edema (arrow). (H&EX200)

G1 showed normal histological structure of gill lamellae and gill arch (Fig. 4a**& b**). Concerning G2, there were sections of parasites between gill filaments (Fig. 4c **& d**), congestion of primary gill lamellae (Fig. 4e), edema, congestion and inflammatory cells infiltration in gill arch (Fig. 4f). G3 showed degenerated parasites between gill filaments (Fig. 4g), lamellar telangiectasis (Fig. 4h), edema and inflammatory cells infiltration in gill arch (Fig. 4i). G4 showed few degenerated parasitic sections with moderate lesions in gill lamellae and gill arch (Fig. 5a and b). G5 showed degenerated parasites, congestion of primary gill lamellae (Fig. 5c) and moderate lamellar telangiectasis (Fig. 5d) with edema, congestion and inflammatory cells infiltration (Fig. 5e). G 6 showed complete absence of parasites (Fig. 5f), mild gill lamellae and gill arch lesions (Fig. 5g **and** Fig. 5h). G7 showed nearly normal gill lamellae (Fig. 6a) with edema in gill arch (Fig. 6b). G8 revealed mild gills lesions (Fig. 6c and d).

### Histopathological lesion scoring

Recorded lesions were scored according to their severity as shown in Table 10.

**Table 10:** Scoring of histopathological alterations in all treated groups

Lesions	G1	G2	G3	G4	G5	G6	G7	G8
Parasites between gill filaments	0	3	2	1	1	0	0	0
Congestion of primary gill lamellae blood vessels	0	3	2	1	1	0	0	0
Lamellar telangiectasis	0	3	2	1	1	1	1	1
Edema of gill arch connective tissue	0	2	2	2	1	1	1	1
Congestion of gill arch blood vessels	0	2	2	2	2	1	0	0
Inflammatory cells infiltration in gill arch	0	2	2	2	2	1	0	0

The score system was designed as: score 0 = absence of the lesion in all fish of the group (n= 5), score 1= (<30%), score 2= (<30% – 50%), score 3= (>50%)

## Discussion

In the present work, we evaluated natural ecofriendly products which are contained compounds as flavonides; saponins; alkaloids; glycosides; etheric oils; many vitamins; minerals; trace elements; polyphenols; tannins and bitter substances. Two compounds were used as Herb-All™ LIVER which improves conversion from feed into energy and appropriate glucose supply. It also; supports the function of the liver and the hepatopancreas, a key organ of the body. This pure herbal mixture can prevent fatty liver disease and improve protein metabolism. It also; improves the excretion of toxins and metabolites from the body. The active secondary plant compounds of Herb-All™ LIVER promote acylation reactions to facilitate conversion of lipids to phospholipids and reduce problems linked to inflammation. In shrimp, Herb-All ^TM^ LIVER protects against acute hepatopancreatic necrosis disease (AHPND).

The second compound was Herb-All™ PARA-X which acts as plant-based protection against various parasites because it strengthens the natural defense mechanisms of the animals. Herb-All™ PARA-X contains herbs with antiparasitic properties to interrupt the reinfection cycle and to keep the parasitic pressure low. With this plant-based solution, feed production needs no separate medicated regimen and there are no withdrawal restrictions. In aquaculture, Herb-All ^TM^ PARA-X controls endo- and ectoparasites naturally. It reduces fish losses and improves the performance of the animals under parasitic pressure.

Our findings showed that infested fish had greater levels of cortisol, glucose, and lysozyme than did healthy fish. It is widely known that the major stress response in teleosts is the release of cortisol from the hypothalamic-pituitary–interrenal (HPI) axis; when the hypothalamic-pituitary-interrenal (HPI) axis is stimulated by a stressor. The stress-induced hyperglycemia (an increase in blood glucose concentration) is then mediated by cortisol, which is assumed to be essential for maintaining the elevated energy requirement linked to stress.

When parasites are present in fish gills, the gill surface area is decreased, the hydromineral balance is upset, ATP-ase expression is elevated, and mitochondria-rich cells undergo apoptosis [39]. Due to this process, the fish receive less oxygen, which results in pale gills, shaky movement, faded colour, stress, and even death [40]; [41]. In addition to gills, the skin is a preferred attachment location for parasites. This is a result of the parasite attaching itself to the host’s gills and skin in order to obtain nutrients for development, such as blood or epidermal cells [42] According to this study, *O. niloticus* blood glucose levels rise when they are infested with light, medium, and heavy category ectoparasites. The blood glucose profile was suggested as a quick way to assess the health of the fish. This result is consistent with another study that found fish infected with parasites did not exhibit any change in haemotological parameters [43].

Most vertebrates, including fish, require glucose, a monosaccharide of the aldohexose group, as a source of energy and carbon. According to [44], blood glucose concentration is a commonly utilized physiological indication that conveys the overall health status of fish.

The main source of carbohydrate energy for vertebrates is glucose, which is primarily stored as the a-linked polymer glycogen in the muscles and liver (also; known as “animal starch”); [45].

Stressor mediates changes in plasma cortisol [46] which stated that plasmatic levels of cortisol were increased quickly after exposure to acute stress and the standard conditions were restored in a few hours. In addition, the response varies depending on the type of stress and the species of fish under investigation.Because fish constantly interact with their surroundings through their skin and gills, water quality—which includes things like salinity, temperature, dissolved oxygen, nitrites, PH, and pollutants—is essential to the health of fish [46]. Though oxygen poses a natural threat to the lives of aerobic species, oxygen in its molecular form, or O2, is necessary for its molecular form, oxygen aerobic organisms which cannot survive without oxygen, despite the fact that oxygen is necessary for several metabolic activities that are critical to their survival. Nevertheless, oxygen poses a constant threat to their survival. Fish have a built-in, powerful antioxidant defense system and, like all aerobic organisms, are vulnerable to the effects of reactive oxygen. Furthermore, it was demonstrated by **Scapigliati**et al. [47]. that low oxygen levels had a detrimental effect on sea bass immunoglobin levels. Numerous metabolic processes that are critical to aerobic life.

According to **Bianca** [46], fish under stress experience both short-term physiological changes (acute stress) brought on primarily by certain hormones and long-term physiological changes (chronic stress conditions) such as loss of scales, fin damage, growth and reproduction, immunity defense, and health. Furthermore, oxidative stress as a welfare metric may offer a fresh method for assessing fish quality [48] **Bagni**et al.., **2007**). Yearling brook trout that had been starved for 12 days had low T3 levels, according to **Higgs and Eales** [49] who also noted that fasting decreased T4 degradation and deiodination. According to **Wang**et al. [50]. T3 stimulates the somato-tropic axis and has been demonstrated to be more responsive to hunger [51].

All groups in the current investigation experienced a significant increase in plasma levels following acute stress exposure. Their coordinated efforts on energy metabolism were reflected in changes in plasma cortisol and thyroid hormone levels [52]. Even though it may be influenced by species, feeding, reproductive cycles, seasonal cycles, photoperiod, husbandry conditions, and sampling, cortisol (measured by RIA) is widely used as a long- and short-term stress condition index [53]. Furthermore, multiple stress conditions appear to amplify the cortisol response [54] **Ortuno**et al., **2002**). Furthermore, cortisol appears to be the primary regulator of mineral regulatory systems in teleostean fish, which lack aldosterone [55].

Immunity components are both innate and adaptive in teleosts. Fish have an innate immune system, just like other vertebrates, which serves as their first line of defense. Adaptive immunity helps to provide a more focused and efficient response to illnesses by generating random and extremely different repertoires of recombinant activation genes (RAGs) encoding T and B-lymphocyte receptors [56]; [57]. Fish innate immune response is heavily dependent on cytokines, which are significant immune system mediators. The chemical IFN-γ, which is part of fish type II IFN, is involved in boosting phagocytic and nitric oxide activities as well as regulating the expression of proinflammatory cytokines like IL-12, IL-1β, IL-6, and TNF-ɑ (58–59).

The production and release of macrophage cytokines can be either up- or down-regulated, or this can have significant consequences on the immune response. Numerous pathogenicity factors specifically target these crucial immune system molecules. The ensuing instances illustrate how specific infections, contingent on their requirements, deregulate cytokine release to facilitate their persistence and propagation. Since cytokines’ main job is to control inflammation, they are essential for controlling the immune system in both health and illness [60]. TNF-α and IL-1β are two examples of the cytokines secreted by macrophages in response to inflammatory stimuli; [61]; [25].

Regarding the histopathological effects of monogeneans on fish, numerous investigations have been carried out. However, there was infrequent data about their effects on stress indices. In the current study, two experimental groups—healthy fish and infected individuals—had their blood parameters, such as cortisol, glucose, and lactate, which were examined [38]; 67.

On a conclusion; the uses of the two products Herb-All PARA-X^®^ and Herb-All CALM^®^ improve the health status of the experimentally infested fishes (*O. niloticus*) with monogenean parasite which treat the fish as well as improve the viability and willingness of the fish.

## Electronic supplementary material

Below is the link to the electronic supplementary material.

Supplementary Material 1

## Data Availability

All data present in the manuscript. The datasets generated during and/or analyzed during the current study are available from the corresponding author on reasonable request.
