# Supplementary material for: Assessment of a new protocol strategy to control the ectoparasitic infestation in Nile tilapia (Oreochromis niloticus) using efficient natural products
Source: BMC Vet Res. 2025 Jan 11;21:15. doi: 10.1186/s12917-024-04387-z (PMC11724587; doi:10.1186/s12917-024-04387-z)
Supplement: Supplementary file 1 — Supplementary Material 1 [file 12917_2024_4387_MOESM1_ESM.docx]

***Table 1: The experimental design for treatment trials with Herb-All PARA-X***

| Groups | Treatment |
| --- | --- |
| Group1(G1) | control negative group :apparent healthy fish receiving an artificial diet without any additives. |
| Group2(G2) | control positive group : fish infested with external parasites( either naturally or experimentally infested) receiving an artificial diet without any additives. |
| Group3(G3) | fish infested with external parasites( either naturally or experimentally infected) receiving an artificial diet mixed with Herb-All PARA-X (1kg/ton). |
| Group4(G4) | fish infected with external parasites(either naturally or experimentally infected) receiving an artificial diet mixed with Herb-All PARA-X (2kg/ton). |
| Group5(G5) | fish infected with external parasites( either naturally or experimentally infected) receiving an artificial diet mixed with Herb-All PARA-X (4kg/ton). |

**Table 2: The next trials concerning Herb-All CALM**

| Groups | Prophylaxis |
| --- | --- |
| Group6(G6) | fish receiving an artificial diet mixed with Herb-All CALM(1kg/ton). |
| Group7(G7) | receiving an artificial diet mixed with Herb-All   CALM   (2kg/ton). |
| Group8(G8) | receiving an artificial diet mixed with Herb-All CALM   (4kg/ton). |

**Feed Conversion Ratio**

| Group | FCR |
| --- | --- |
| G1 | 2.9 |
| G2 | 3.1 |
| G3 | 3.0 |
| G4 | 2.7 |
| G5 | 2.7 |
| G6 | 2.5 |
| G7 | 1.97 |
| G8 | 1.72 |

**Mortality rate**

| Groups | No.of total fish | No.of dead fish^*^ | Mortality rate (%)^#^ |
| --- | --- | --- | --- |
| Group1 | 30 | - | - |
| Group2 | 30 | 15 | 50 |
| Group3 | 30 | 10 | 33.3 |
| Group4 | 30 | 6 | 20 |
| Group5 | 30 | - | - |
| Group6 | 30 | 4 | 13.3 |
| Group7 | 30 | 1 | 3.33 |
| Group8 | 30 | - | - |

- No.of dead fish during the experiment from 1^st^  to 4^th^ week.

# Mortality rate at the end of the experiment.
